# Role of MRI as a problem-solving tool in screening assessment

**DOI:** 10.1186/bcr2983

**Published:** 2011-11-04

**Authors:** RK Locke, G Rubin

**Affiliations:** 1 Park Centre for Breast Care, BSUH NHS Trust, Brighton, UK

## Introduction

In the UK, MRI is mostly used to stage needle biopsy proven cancer. Less often it is used in problem-solving when there is no proven malignancy. We reviewed its use as a problem-solver after mammographic screening.

## Methods

NHS screening patients who attended assessment between December 2008 and July 2011 and had contrast-enhanced MRI within 3 months were identified. Fourteen out of 47 did not have the MRI as staging, and were reviewed.

## Results

(1) Mammography, ultrasound and/or core biopsy pathology were felt to be discordant in seven cases. MRI confidently excluded malignancy in five. Two had surgery, finding a complex sclerosing lesion in one and a reactive lymph node in the other. (2) Clinical concern was not matched by mammography and ultrasound in four cases. MRI confidently excluded malignancy in three but found an otherwise occult cancer in the fourth. (3) Core biopsies were technically difficult in two cases. MRI confidently excluded malignancy in one and the other was benign on excision. (4) One patient had proven cancer in one breast not needing staging. Screening also showed microcalcifications with a possibly nonrepresentative normal core biopsy in the other breast. MRI was suspicious and this was also cancer at diagnostic surgery.

## Conclusion

MRI, used selectively, was useful in problem-solving at screening assessment. When it was negative, this meant some patients could be reassured without surgery. It was useful where there was clinical suspicion but normal conventional imaging.

